# Rapid reagent free COVID19 detection using MEMS based FTIR spectroscopy and machine learning in NIR and MIR regions

**DOI:** 10.1038/s41598-025-20473-0

**Published:** 2025-10-07

**Authors:** Ahmed Abdelkhalik, Mazen Erfan, Bassem Mortada, Mohamed Gaber, Shereen Saeed, Ghada Ismail, Ahmed ElShafei, MennaAllah S. Mohamed, Bassam Saadany, Yasser M. Sabry, Diaa Khalil

**Affiliations:** 1 Si-Ware Systems, 3 Khaled Ibn El-Waleed Street, Heliopolis, Cairo, Egypt; 2 Faculty of Engineering, ECE Department, Ain Shams University, Cairo, Egypt; 3 Reference Laboratory of the Egyptian University Hospitals, Cairo, Egypt; 4 Faculty of Medicine, Clinical Pathology department, Ain shams University, Cairo, Egypt; 5 Modern University for Information and Technology, Cairo, Egypt

**Keywords:** ATR, COVID-19, MEMS FTIR spectrometer, MIR spectroscopy, NIR spectroscopy, PCR, Infectious disease, Biomedical engineering, Electrical and electronic engineering, Viral infection, Population screening

## Abstract

This study presents rapid, reagent-free detection of COVID-19 using miniaturized MEMS-based Fourier-transform infrared (FTIR) spectrometers integrated with machine learning models. Two portable spectrometers analyze 363 nasopharyngeal swab samples stored in viral transport medium (VTM). The first spectrometer covers the near-infrared (NIR) region (1.3–2.6 μm) and second spectrometer extends from the near infrared to the mid infrared (MIR) region (1.75–4.0 μm). The NIR system uses a transmission configuration, while the one extended to the MIR performs attenuated total reflectance (ATR) measurements on both wet and dried samples. Spectral data undergo preprocessing and analysis using interval partial least squares discriminant analysis (iPLS-DA), with model training and evaluation conducted through Monte Carlo cross-validation. The MIR wet sample model achieves a diagnostic performance with 79% accuracy, a 98% sensitivity, and an area under the curve (AUC) of 0.8. The MIR dry sample model achieves an 80% accuracy and an AUC of 0.79, while the NIR model reaches 66% accuracy and an AUC of 0.64. Spectral features appear in the Amide A and B regions in the MIR range, and in the C–H overtone bands in the NIR range. The full measurement process, including sample handling, completes in under six minutes, supporting its suitability for real-time, point-of-care (POC) testing.

## Introduction

The severe acute respiratory syndrome coronavirus 2 (SARS-CoV-2) also known as COVID-19 has laid deaths to over 13 million people worldwide in 2020 and 2021^1^, and has been declared a global pandemic ever since the beginning of its widespread in 2020. As of 2022, the coronavirus infection and death rate is declining with only seasonal waves of new strains, with the most widespread strain being the omicron BA.5^2^. As the virus is still circulating and evolving, the possibility of a dangerous strain is still present, hence the WHO recommendation of continued monitoring of the virus widespread and the enhancing of testing and vaccines^3^.

The golden standard for the testing of the COVID-19 is currently the Reverse transcription-Polymerase Chain Reaction (RT-PCR) test, which has a long turnaround time of a few hours up to a day before the results delivery^4^, which makes it non-suitable for rapid screening and Point of Care (POC) testing. COVID-19 testing technologies has seen tremendous development in the past years in order to comply with the WHO guidelines where the tests are becoming faster and cheaper. Some of the recent technologies includes ID NOW isothermal amplification process which can deliver results within 15 min^5^. However, it is still limited by the need of trained doctors doing the tests, as well as the high price mark, which only allows its use in necessary rapid tests and not suitable for mass screening. Other testing methods such as antigen and serology have a lower cost, but suffer from lower accuracy and can only detect the virus in its later stages, hence these methods cannot be used in the early detection of the virus^6^. The RT-PCR test is most commonly carried out using viral transport medium (VTM) in which a swab containing the biological material from a patient nasopharynx and oropharynx is submerged to test for the virus existence. This combination has been reported to give high accuracy in the detection of the virus hence its widespread use in COVID-19 detection^7^.

Fourier Transform Infrared (FTIR) spectroscopy is a non-destructive analysis method that has been used in the identification and detection compounds and has long been employed in the identification of pharmaceutical compounds^8^. FTIR spectroscopy has recently gained high attention in clinical studies due to its accuracy, fast analysis of materials and with latest developments in the technology allowing smaller portable devices to be used in the field. Infrared spectroscopy has been used in the detection of multiple diseases including cancer^9^, Malaria^10^, leukemia^11^, HIV^12^, and hepatitis C^13^ becoming a novel tool for POC testing. Hence, its performance in the detection of the COVID-19 virus in clinical settings needs to be evaluated. Wood, Bayden R., et al.^14^ recently demonstrated FTIR spectroscopy capability in the detection of COVID-19 in saliva samples where it achieved 93% sensitivity and 82% spcificity, saliva samles were air dried on a BaF_2_ window before being placed on the benchtop spectrometer which takes up to 15 min, making it less suitable for POC Mass screening. Other body fluids such as blood plasma was investigated by Calvo-Gomez, Octavio, et al.^15^ and has shown FTIR spectroscopy capability of detecting COVID-19 with 94.55% sensitivity and 98.44% specificity using blood samples after being centrifuged to separate the plasma for scanning, yet such study was limited by the time of centrifugation and the use of a benchtop spectrometer, which was less suitable for POC mass screening. Another study for FTIR detection using blood plasma was conducted by Zhang, Liyang, et al.^16^, and they achieved 83% sensitivity and 98% specificity, the capability of using the same VTM sample in both FTIR and PCR analyses was previously investigated by Nogueira, Marcelo Saito, et al.^17^, and achieved 78% accuracy, in that study, samples were dried on an aluminum foil in laminar air over a 2 h duration, and the viral content of the samples could have decreased during the drying process.

Miniaturized MEMS based FTIR spectrometers are novel instruments that has been employed in feed testing^18^, environmental pollution monitoring^19^ and biomedical studies including skin analysis^20^, and has shown its effectiveness in the analysis of biofluids^21^, making it a very attractive low cost device for mass screening. However, MEMS FTIR spectroscopy scope of analysis is limited by the capabilities of the device in terms of signal-to-noise ratio, spectral resolution and range of wavelengths detectable especially when using uncooled optical detector^22^.

**Fig. 1:**
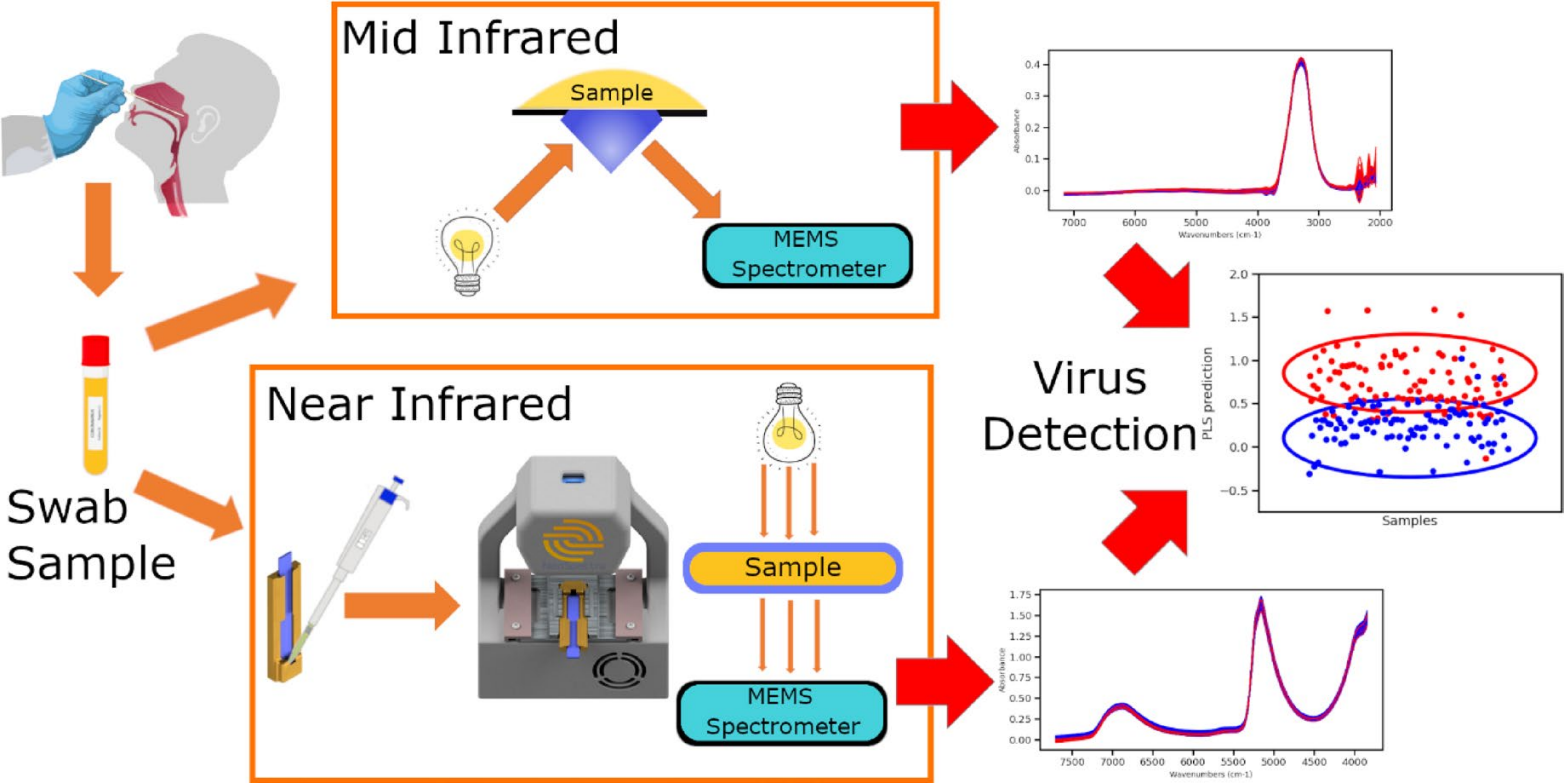
Measurement and Analysis steps for COVID detection, samples are scanned with MIR ATR device and NIR transmission device, and a machine learning model is developed to use the measured spectrum for detection.

In this work infrared spectroscopy using miniaturized microelectromechanical systems (MEMS) spectrometer^23–26^ is evaluated as a potential tool for COVID-19 detection, given the limited performance of the MEMS device compared to benchtop devices on the expense of being portable and cheap enabling mass screening. Two different spectrometer devices are used, with the aim of maximizing the limit of detection for the different sample contents as shown in Fig. 1. Transmission spectroscopy is used in the near infrared (NIR) region, while attenuated total reflection (ATR) is used in the mid infrared (MIR) region. Since organic materials and biofluids contain many functional groups, each contributing to the overall spectrum in the infrared region, hence the interpretation of the obtained spectrum by inspection is not practical. It is also not ideal for discriminant machine learning models to work on a large number of variables with countless sources of variability, hence in this study principle component analysis methods are employed in the interpretation of the measured spectrum and in obtaining the statistical performance of infrared spectroscopy in the detection of the COVID-19 virus.

The rest of this article is organized as follows.

- The Methods section explains the sample collection procedure, sample statistics, the scanning devices, the scanning protocol and the spectra processing techniques.
- The Discussion section contains a detailed analysis of the measured spectrum and the performance of the predictive models based on the collected samples.
- The Conclusion section summarizes the results and lists the important conclusions then introduces points of study for future work.

## Methods

### Sample collection

Nasopharyngeal swab samples were collected from individuals undergoing RT-PCR testing at the Reference Laboratory for Egyptian University Hospitals (RLEUH). Each participant provided written informed consent, acknowledging that their samples would be used for clinical research in addition to routine RT-PCR testing. The swabs were collected by trained hospital nurses and placed in 1.5 mL of VTM to preserve viral integrity over time. Within a few hours of collection, the samples were processed using RT-PCR. A balanced subset of both positive and negative samples was then selected for optical spectroscopy analysis, conducted within 12 h of receiving the PCR results. The study was submitted to the Ethics Committee at the Supreme Council of University Hospitals in Egypt and was evaluated by the ethical committee and the study was approved. All methods were carried out in accordance with relevant guidelines and regulations.

In parallel, samples were analyzed using the FTIR spectrometer within a maximum of 24 h from the time of collection. During this period, the vials containing the VTM were stored at room temperature. It has been demonstrated that samples preserved in VTM remain stable for up to three days at room temperature without significant degradation in viral load^27^. Therefore, conducting the optical spectroscopy analysis within 24 h ensured comparable viral loads between RT-PCR and FTIR measurements.

### Samples statistics

Between January 11 and May 7, 2022, a total of 162 samples were analyzed using the NIR spectrometer and 201 samples using the MIR spectrometer, including both wet and dried samples. Samples were randomly selected from the VTM pool collected daily, with no restrictions on the health status or age of the participants. The dataset for each device was collected in a period of only 2 weeks per device, hence one can rule out any aging or drift in the measurement device. The rest of the study duration was devoted to the development and optimization of the 3 different devices used in the study.

In the NIR dataset, 26 samples were identified and excluded as outliers using the Isolation Forest algorithm^28^. The remaining 136 samples—comprising 68 COVID-19 positive and 68 negative cases—were used to construct the PLS model for the near-infrared region in the exploratory analysis. In the MIR dataset, 43 outliers were similarly excluded. Among the remaining samples, the wet subset consisted of 40 positive and 57 negative samples, while the dried subset included 25 positive and 36 negative samples. These were used in building the PLS model for the mid-infrared region.

**Fig. 2:**
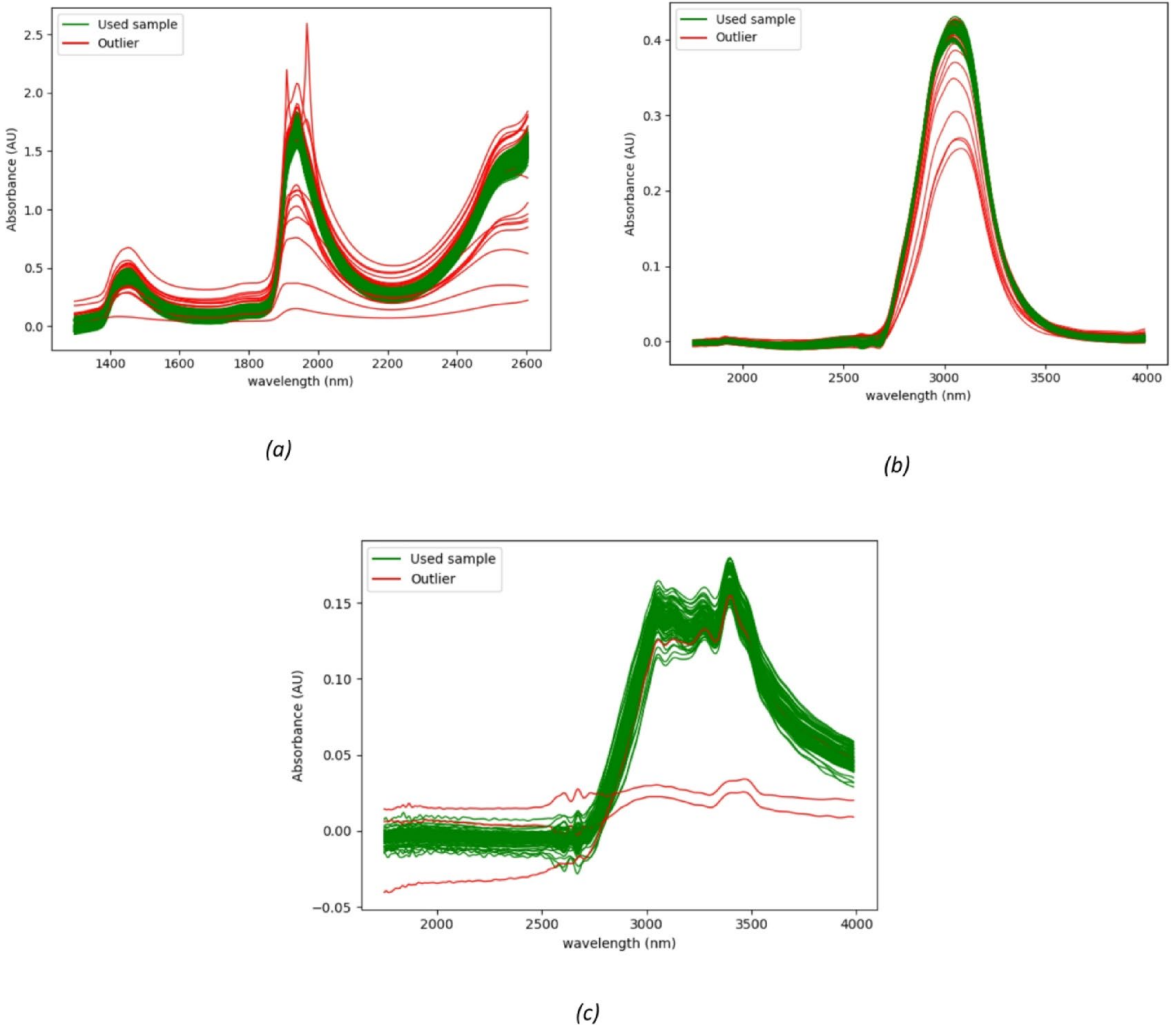
Plots for the outliers and used samples. (**a**) Near infrared outlier spectra showing that outliers have lower absorbance at 1900 nm. (**b**) Mid infrared outlier spectra showing that outliers have lower absorbance at 3000 nm. (**c**) Mid Infrared dried outlier samples showing that outliers have lower absorbance at 3400 nm.

Outlier detection in the NIR measurements was largely attributed to the presence of air bubbles which significantly reduced the sample absorbance and the presence of mucus other some samples which increased the absorbance significantly across the entire spectral range. In contrast, outliers in the MIR measurements were primarily linked to the sample not covering the ATR crystal completely. Other issues were related to the cleaning protocol, as described in detail in the scanning protocol section for each device. Representative spectra of the outliers in all three cases are presented in Fig. 2.

It is clear that all outlier samples can be easily detected by inspecting the spectrum or by automated algorithms. The source of outliers is different from one set of samples to another such as the presence of air bubbles or mucus in case of NIR transmission device. A fault in sample insertion onto the MIR ATR device can be amended by acquiring another sample from the VTM vial which can be done on-site. The presence of outlier samples can be reduced in the future by automating the insertion of samples into the device.

### Scanning devices and protocol

#### NIR scanning

As the study focused on detection using collected samples without additional preparation steps, the VTM in which the swabs were stored was deemed the most suitable candidate for scanning due to its consistent and repeatable spectral profile. The absorption characteristics of VTM in the NIR region closely resemble those of water. Based on this, a transmission path length of approximately 0.3 mm in water was calculated to provide the optimal trade-off between signal-to-noise ratio and absorbance across the full spectral range. Consequently, 0.3 mm-thick cuvettes (Vitrocom) were used to hold the samples during scanning with the NIR spectrometer.

**Fig. 3:**
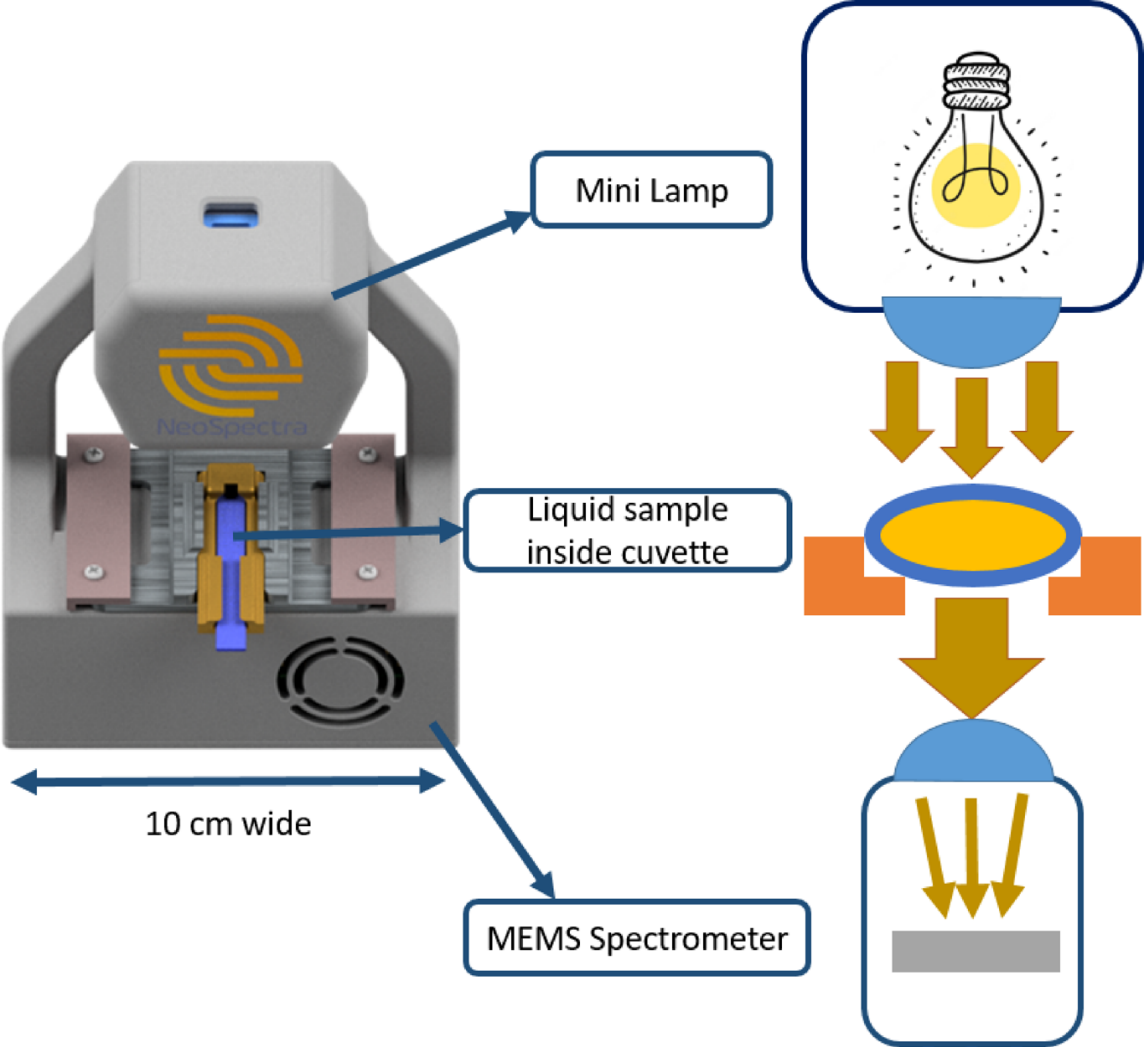
Near Infrared portable Scanner, light transmits through the sample then hits the MEMS based spectrometer. Samples in a cuvette are inserted using a disposable holder for ease of insertion and reduce contamination.

The NIR spectrometer used in this study is a free-space-coupled, MEMS-based FTIR spectrometer. The optical core—comprising the MEMS chip, micro-optic mirrors, and an uncooled extended InGaAs detector—is assembled into a tiny package. The internal components of the spectrometer are discussed in detail in a separate publication^25^. A 5 × 10 × 13 cm³ metallic housing was designed to secure the light source and the cuvette fixture in precise alignment with the spectrometer, as illustrated in Fig. 3. This miniaturized design enabled the entire measurement setup to fit comfortably in the palm of a hand. The device was compact, handheld, and robust, with high resistance to mechanical vibrations. The spectrometer covered the extended NIR spectral range from 1.3 to 2.6 μm and operated at a spectral resolution of 66 cm⁻¹ (equivalent to 16 nm FWHM at 1550 nm), which was selected to optimize the signal-to-noise ratio. Higher spectral resolution would have reduced this ratio.

For each measurement, 45 µL of VTM was drawn from the sample vial and transferred into the cuvette using a disposable holder. The cuvette and holder were then inserted into the spectrometer fixture, where light passed through the sample and the transmission spectrum was recorded. After each measurement, both the cuvette and holder were disposed of in a chemical waste container to prevent contamination. The complete measurement process is shown in Fig. 1, and a detailed view of the device is presented in Fig. 3.

#### MIR scanning

For wet samples scanned in the MIR region, the VTM spectrum is dominated by strong water absorbance, which restricts measurements to attenuated total reflectance (ATR) mode without prior drying. The MIR measurement system employed a MEMS-based, free-space FTIR spectrometer as well but integrated with an uncooled PbSe detector. The spectrometer, along with its associated electronics, was enclosed in a metallic housing. To enhance the mechanical stability. The entire setup setup—including the spectrometer, light source, and ATR accessory (Quest, Specac)—was mounted on an Acrylic board. The ATR accessory used a single-reflection diamond crystal for sampling. The spectrometer was capable of capturing spectra between 1.4 and 4.8 μm (2070–7150 cm^−1^), which includes the Amide A region, among other biologically relevant bands. All measurements were performed at a spectral resolution of 66 cm⁻¹.

**Fig. 4:**
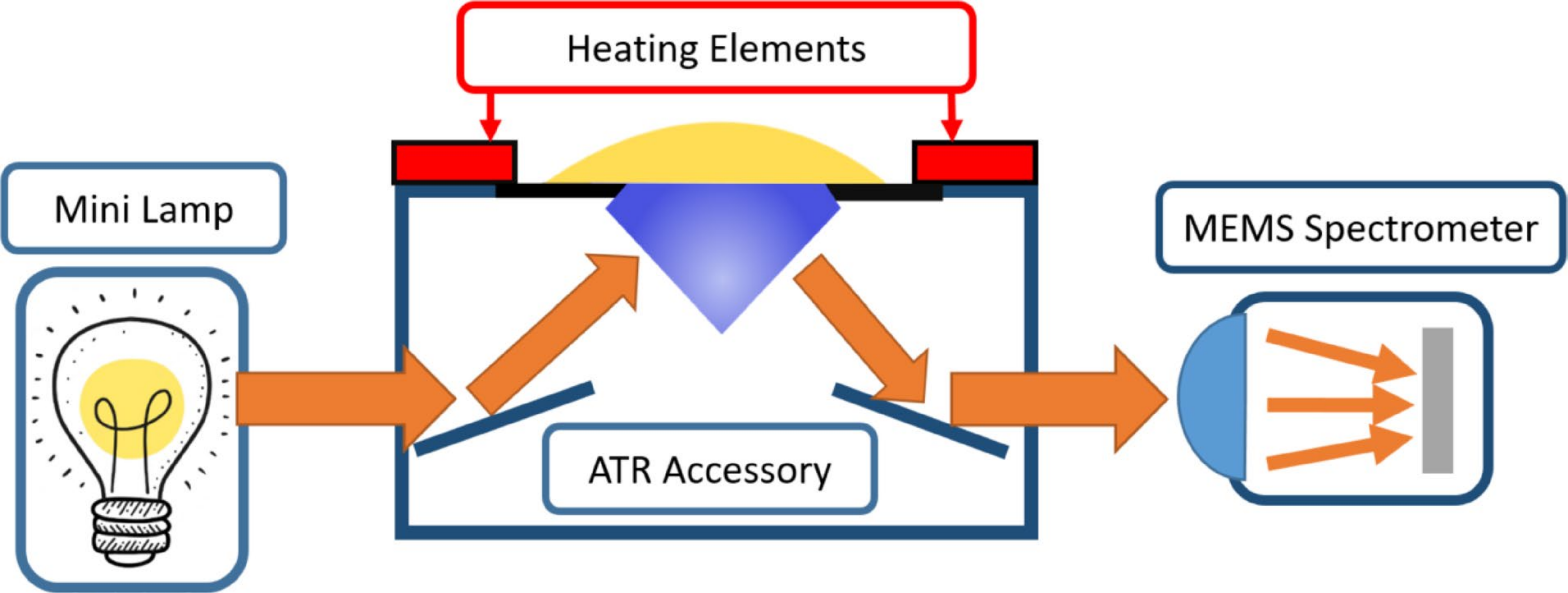
Mid Infrared Scanner, samples are deposited onto the ATR crystal, and light that interacts with the sample is directed into the MEMS based spectrometer.

A 10 µL aliquot of each VTM sample was drawn and deposited directly onto the ATR crystal. The measurement setup is illustrated in Fig. 4. The entire measurement process took approximately six minutes per sample. Of this, two minutes were allocated to spectral acquisition to maximize the signal-to-noise ratio (SNR), followed by a cleaning procedure. After each measurement, the ATR crystal was cleaned with 70% alcohol using a cotton swab. Once the alcohol had evaporated, the crystal was wiped with lint-free wipes (Kimtech) dampened with distilled water to eliminate any potential viral contamination. The device was then left to air dry for an additional two minutes to ensure complete evaporation of any residual alcohol or water. Before the next sample was applied, a new background spectrum was recorded to confirm that the ATR crystal was thoroughly cleaned and free of contaminants. In some cases, residual alcohol left a distinct spectral signature in the recorded data. These samples were excluded from the study. Based on these observations, a two-minute evaporation period was determined to be sufficient for eliminating any alcohol-related spectral interference.

#### MIR dried samples scanning

To enable high-throughput scanning of dried samples, the metallic housing of the diamond ATR crystal, along with the surrounding metal components, was heated to a constant temperature of 75 °C for one minute. This elevated temperature was verified to cause only viral inactivation^29^, which did not affect the spectroscopic results, as no biological amplification occurs during FTIR measurements. For dried sample acquisition, the spectrometer resolution was increased to 45 cm⁻¹ to better resolve the more complex spectral features of dried samples. The smaller spectral resolution causes an increase in the noise in the spectrum, therefore to compensate for the reduction in SNR, the scan time was increased to compensate this change. It is worthy to note that the SNR is inversely proportional to the resolution. The scan time was increased from 2 min to 3 min since the SNR is proportional to the square root of the scan time^30^.

A 7 µL aliquot of each VTM sample was placed onto the heated ATR crystal and allowed to evaporate over one minute. A second 7 µL aliquot was then applied to the same location to enhance measurement repeatability. The volume of the aliquot was limited to 7 µL to confine the VTM precipitation to the surface of the crystal and prevent the “coffee ring” effect^31^ from pushing the precipitate out of the crystal. While using 2 drops had the highest spectrum repeatability with the sample being uniformly distributed on the crystal, adding a third drop did not affect the repeatability. The sample was left on the crystal for a total of five minutes while the spectrometer continuously recorded spectra throughout the process. It was observed that the recorded spectra exhibited some drift during the first two minutes of measurement, likely due to thermal effects on the VTM. After this initial period, the spectra are stabilized. The stabilized portion of the spectra during the last 3 min was used for constructing the PLS model.

#### Data preprocessing and analysis

Data analysis and model construction were done using the Scikit-learn package in Python. For the NIR data, to reduce the uncorrelated variance in the samples, the spectrum recorded within the two minutes was first averaged, then the spectrum of the empty cuvette before the sample insertion was used as a background to account for absorption differences between the different cuvettes. The resulting spectra were then used to construct an Interval Least Squares Discriminant Analysis (iPLS-DA) model.

For the MIR data, to reduce the uncorrelated variance in the measurements, the region from 4 to 4.8 μm was removed due to the detector low SNR in this region and the existence of the carbon dioxide peak, which was occasionally present in some of the measurements. The region of 1.4–1.75 μm was also excluded due to low SNR and overlap with the NIR spectrometer’s range, where higher quality data were already obtained. As a result, the analysis focused on the region 1.75 μm to 4 μm, which contains the amide A region, and an iPLS-DA model was constructed using the same method discussed for the NIR data. For the wet samples, spectra collected over the two-minute scan were averaged to improve the SNR prior to modeling, The scan time was increased to three minutes for dry samples to compensate for the difference in the SNR.

#### iPLS modeling

The constructed model is based on interval-PLS (iPLS)^32^, which applies a non-linear iterative partial least-squares method (NIPLAS) to obtain the principal components in the data that correlate to the prediction. This transfers the data from a high dimensional space to a low dimensional space, after which a linear regression model is built on the low transformed data to avoid overfitting.

The iPLS algorithm initially splits the spectrum into 10 equal regions. A PLS model is constructed for each region individually, as well as for combinations of different regions, until the optimal spectral interval and number of principle components number are identified using cross validation, The MIR dry samples spectrum is divided into 20 regions instead of 10 to account for the increased resolution, and to allow finer separation between distinct spectral peaks. The initial use of 10 regions was chosen arbitrarily.

Different combinations of preprocessing were applied to the data and the best performing preprocessing method was chosen during hyperparameters selection specifically first derivative transformation, multiplicative scatter correction (MSC), detrending and standard normal variate (SNV) normalizations^33^^34^,. The selected preprocessing methods greatly reduce the light path length variance in the sample, while preserving localized features in the spectrum. The MSC algorithm was performed on all spectra using a reference spectrum generated from the training set to avoid data leakage. This means that the MSC algorithm uses the average of only the training set as a reference, therefore, it does not leak information from the evaluation set into the training set.

In order to obtain an accurate estimate of the accuracy of the devices and the modelling scheme in the detection of the virus, an evaluation analysis was carried out using nested cross-validation on the original data before outlier removal. Stratified K-fold splitting initially splits the data into training and evaluation sets. Then an outlier removal model based on the isolation forest algorithm is fitted and applied to the training set and the resulting model is used to remove outliers from the evaluation set. After that the training set is once again split into training and cross-validation sets using an inner stratified K-fold cross validation to determine the optimal model hyperparameters. The hyperparameters include the optimum range, PLS components and the preprocessing. The trained iPLS model accuracy is reported over the remaining samples in the evaluation set. This process is repeated using the different folds as the evaluation set and the combined results are used as an unbiased estimate of the device’s accuracy.

Stratified K-folds cross validation splits the positive samples into K groups, and the negative samples into K groups, then one positive and negative group is used for evaluation and the other K-1 groups are used for training, then the process is repeated with the other positive and negative groups, and the results are aggregated for all K groups being used for evaluation. The number of folds is 10 for NIR and MIR wet samples and 5 folds for dry samples due to the smaller number of samples in the dataset.

An exploratory analysis using cross validation was done to determine the important regions in the spectrum for the virus detection. Monte Carlo cross validation was used in this analysis which picks one positive and one negative samples randomly to be used for cross validation in each iteration, this is repeated over 100 iterations, then the results from all iterations are aggregated to produce the result for a specific choice of model hyperparameters and preprocessing and wavelength region in the iPLS analysis, then the regions with the highest accuracy were determined and are shown in the discussion section, the applied cross-validation method produces results that are not biased by the unequal number of positive and negative samples in the dataset, however while this analysis provides insight into the data and the important regions for the virus detection, it provides a biased estimate of the device’s accuracy because this method picks the regions that maximizes the cross validation accuracy.

## Discussion

**Fig. 5:**
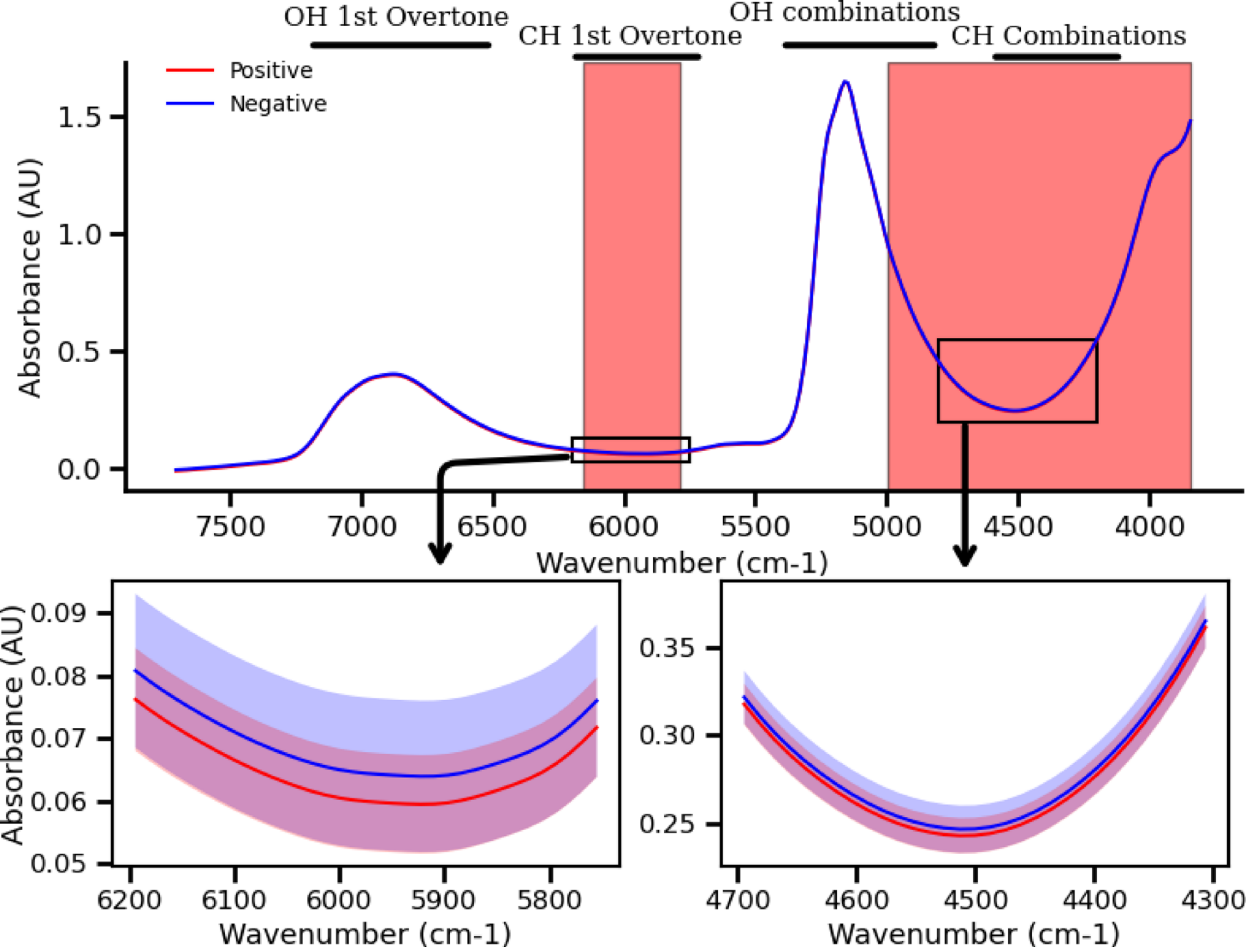
Average and standard deviation of the NIR spectrum of positive and negative VTM samples with important regions annotated, 1 st CH overtone and CH combinations regions show as determining regions in the detection.

Figure 5 shows the mean absorbance spectrum of positive and negative patients as well as the standard deviation spectra of the NIR samples. It is clear that the positive samples have lower absorption around the 5500–6500 cm^−1^ region, which corresponds to the first overtone of C-H stretching vibrations, this region was shown to have high accuracy in the virus detection by the exploratory analysis. The combination band region of C-H tones also showed reduced absorption in positive samples, which agrees with the choice of highly discriminant spectral regions obtained by the exploratory analysis. This suggests a possible correlation with the virus or associated biological material containing C-H bonds. Despite the subtle differences between the measured spectra of positive and negative samples, these variations are detectable using our system’s SNR, even accounting for the measurement variance.

**Fig. 6:**
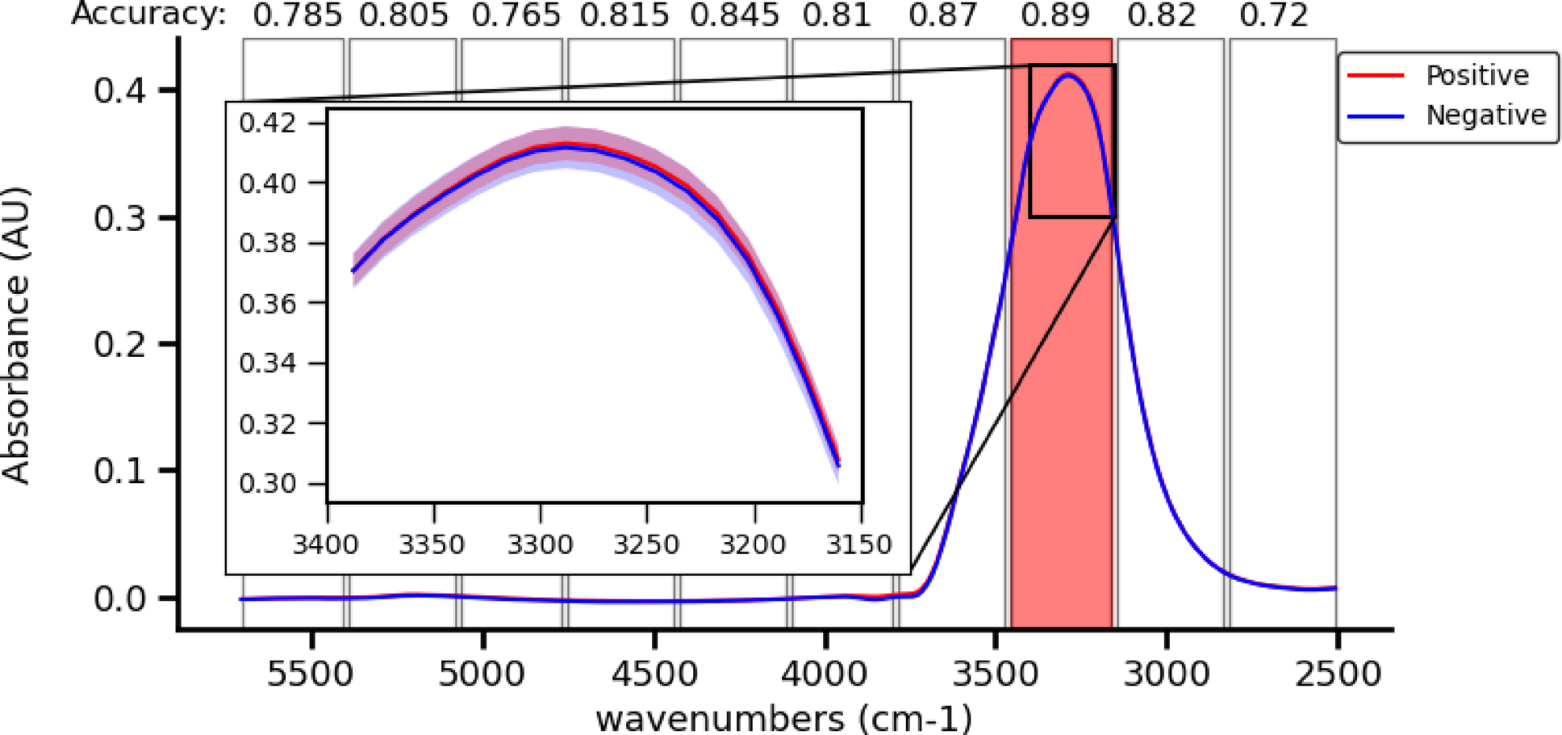
Average and standard deviation of the MIR spectrum of positive and negative VTM samples with the cross validation iPLS accuracy of each region from the exploratory study shown at the top, the protein peaks at 3300 cm^−1^ show the best accuracy in the detection.

Figure 6 shows the mean and standard deviation spectra of positive and negative wet samples in the MIR region. There is a distinctive increase in Amide A absorption around the 3000–3500 cm^−1^ region, which is clearly notable in the low wavenumber region of the peak. The feature around 3500–4000 cm^−1^ is attributed to variations in diamond ATR crystal absorption, as it showed low correlation with sample class in the iPLS exploratory analysis. External effects were minimized in this study by ensuring that an equal number of positive and control samples are scanned each day. Additionally, all samples scanned on the same day were drawn from the same VTM batch and stored under identical conditions until measurement.

**Fig. 7:**
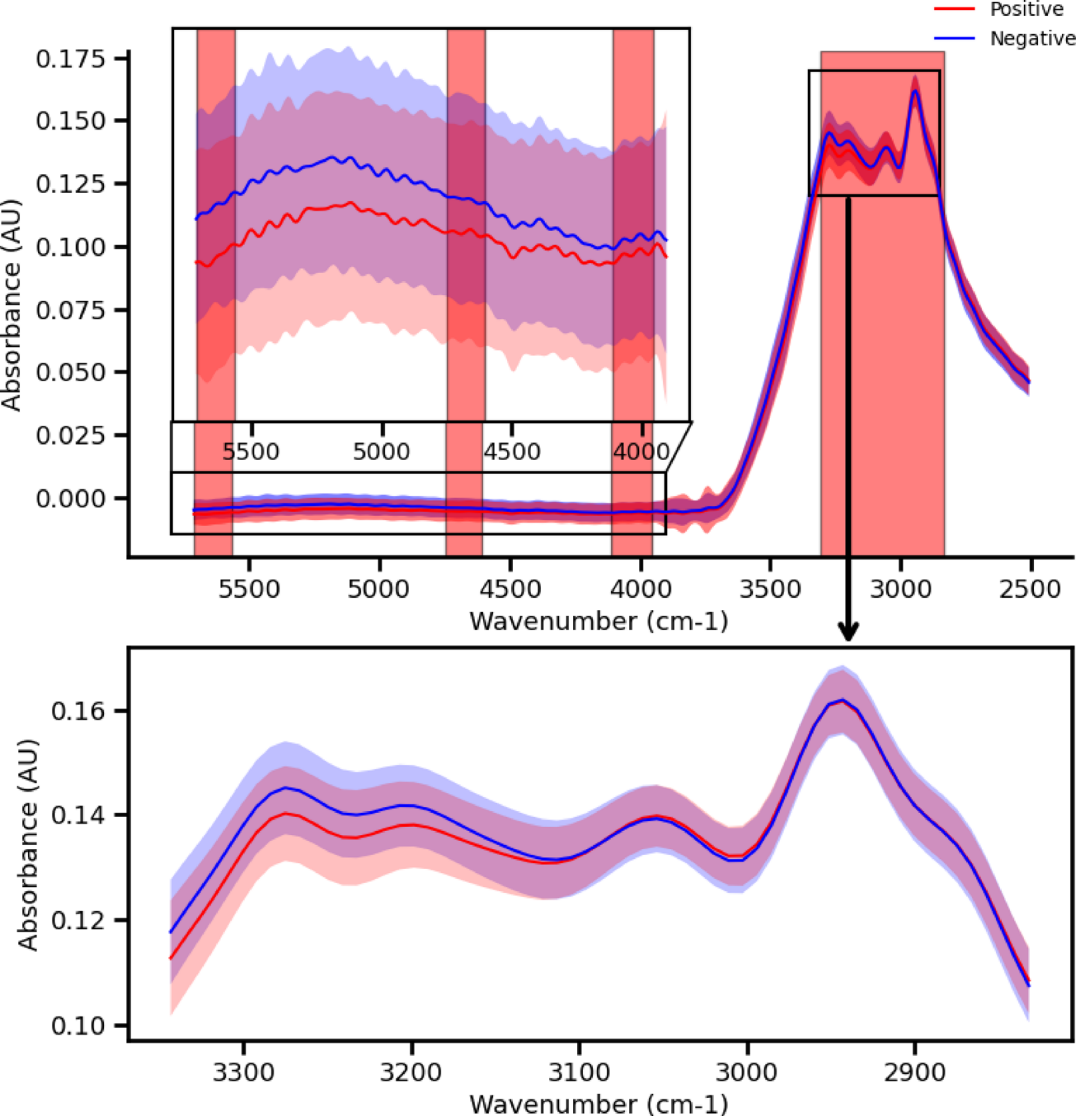
Average and standard Deviation of the MIR spectrum of positive and negative dried VTM samples, the 3300 cm^−1^ region shows the highest potential in the detection.

Figure 7 shows the mean and standard deviation spectra of positive and negative dry samples in the MIR region. The variance of the measured samples is an order of magnitude larger than that of the wet samples, which might be caused by the less sample homogeneity as opposed to wet samples. The most notable features are between 2800 and 3400 cm^−1^ which includes the Amide A and OH bond (3200–3400 cm^−1^) and Amide B (3070 cm^−1^) regions, as well as the aliphatic region (2930 cm^−1^), with a small increase in the Amide B region and a large decrease in the Amide A region for positive samples. In addition, there is a decrease in the absorption of the positive samples across the entire measured near infrared region (4000–5700 cm^−1^).

### iPLS model result and discussion

**Fig. 8:**
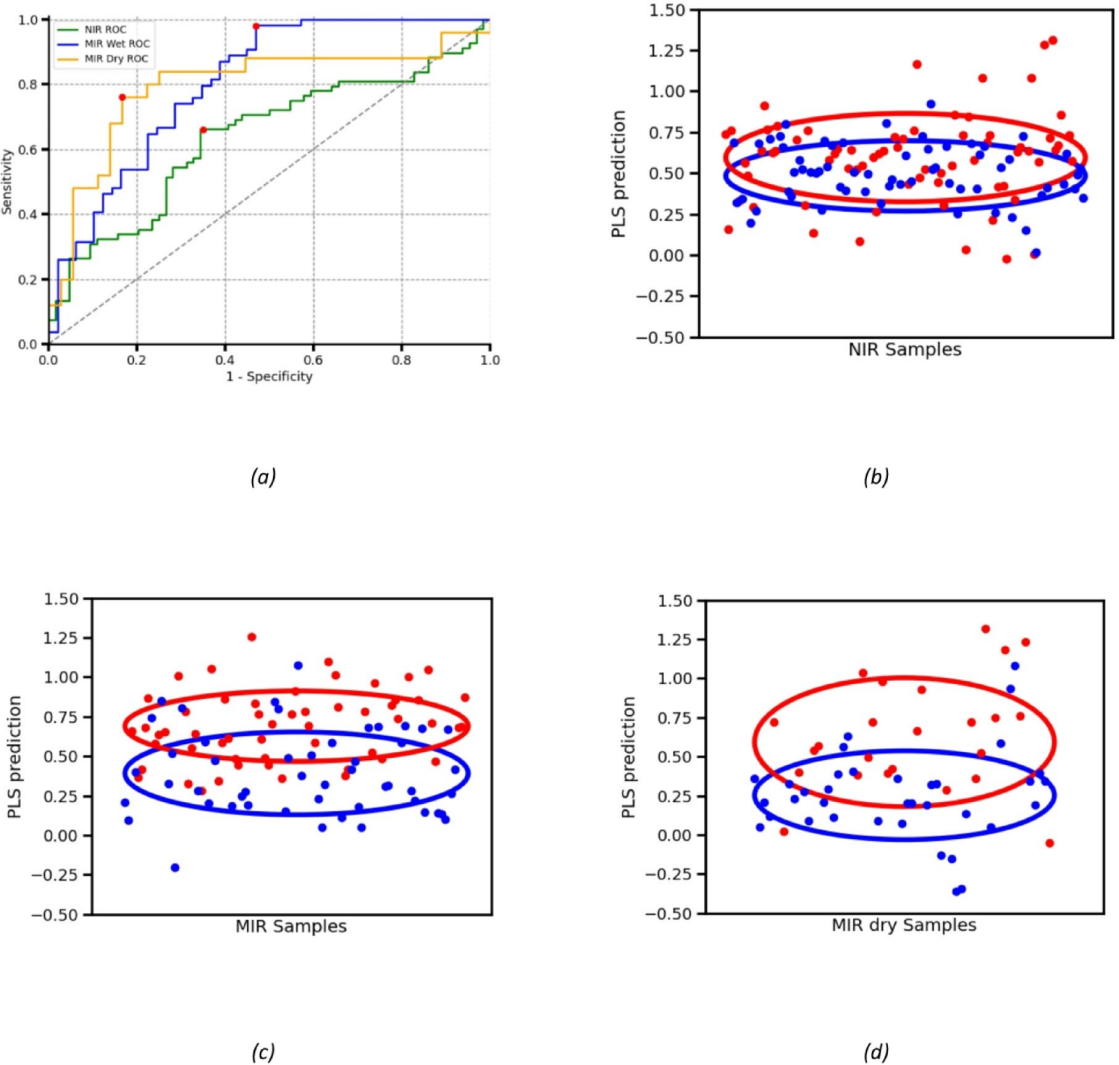
(**a**) ROC curve for the performance of NIR and MIR spectrum models, highest accuracy points are highlighted in red and shown in Table 1. iPLS predictions of the 3 models based on (**b**) NIR, (**c**) MIR & (**d**) Dried MIR samples spectra. The mean and confidence intervals are outlined, it is clear that mid infrared models have better accuracy than the near infrared model.

For the NIR data, the evaluation analysis using the iPLS model achieved an accuracy of 66% (± 8% at 95% confidence interval), with 66% sensitivity, 65% specificity, and an area under the curve (AUC) of 0.64. The cross-validated receiver operating characteristic (ROC) curve is shown in Fig. 8 (a). This result is expected, as the NIR region primarily contains overtones of fundamental molecular vibrations, rather than the stronger fundamental absorbance bands.

For wet samples in the MIR region, the iPLS-based model achieved an accuracy of 79% (± 8.3% at 95% confidence interval), with 98% sensitivity, 53% specificity, and an AUC of 0.8. The exploratory iPLS analysis selected the Amide A region as the most discriminative for COVID-19 detection. Models built using other regions, as illustrated in Fig. 6, did not yield comparable performance. However, reliance on a single spectral region suggests the model may have limited ability to distinguish COVID-19 from other infectious diseases that also impact the Amide A absorption.

For dry MIR samples, the PLS model achieved an accuracy of 80% (± 7.5% at 95% confidence interval), with 76% sensitivity, 83% specificity, and an AUC of 0.79. The performance difference between wet and dry samples falls within the 95% confidence interval, indicating that both formats offer comparable predictive capability for COVID-19 detection based on the collected data.

However, the analysis of dry samples is constrained by higher spectral variance, primarily attributed to the composition of the VTM. Thus, the use of an alternative transport medium with a more consistent spectral profile could further enhance the reliability of dry sample analysis.

Table 1 summarizes the performance estimates for each device in different IR regions and the model predictions for individual samples are shown in Fig. 8 (b), (c) and (d). Figure 8 (a) shows the ROC curves across the NIR and MIR regions, with the MIR data demonstrating superior predictive power. This is primarily due to the more pronounced absorbance changes in the Amide A region compared to its weaker overtone signals in the NIR. Table 2 shows the cross validation scores from the exploratory analysis showing the ultimate performance if number of samples was significant.

**Table 1:** iPLS models performance on the evaluation dataset for the investigated devices covering different infrared regions.

Device	Accuracy ± 95% CI	Sensitivity TP/(TP + FN)	Specificity TN/(TN + FP)	AUC	Number positive	Number negative	Wavelength range (nm)
NIR	66% ± 8%	66%	65%	0.64	68	64	1300–2600
MIR Wet	79% ± 8.3%	98%	53%	0.8	54	49	1750–4000
MIR Dry	80% ± 7.5%	76%	83%	0.79	25	36	1750–4000

**Table 2:** iPLS exploratory analysis cross validation scores of the models performance for the investigated devices.

Device	Accuracy ± 95% CI	Sensitivity TP/(TP + FN)	Specificity TN/(TN + FP)	AUC	Number positive	Number negative	Wavelength range (nm)
NIR	77% ± 6%	72%	81%	0.79	68	68	1300–2600
MIR Wet	89% ±4%	83%	95%	0.93	40	57	1750–4000
MIR Dry	86% ± 4.6%	85%	86%	0.89	25	36	1750–4000

## Conclusion

The presented research demonstrates the viability of using portable optical spectroscopy for the detection of COVID-19, employing affordable, small-form-factor equipment suitable for point-of-care (POC) testing. The entire sampling and scanning process takes less than six minutes, enabling its potential application in mass screening scenarios. Among the spectral regions studied, the mid-infrared (MIR) region emerged as the most effective for COVID-19 detection, achieving an accuracy of 80%. In contrast, the near-infrared (NIR) region demonstrated approximately 14% lower accuracy. Distinct spectral features were identified in both regions: in the NIR, relevant features appeared in the C–H first overtone and combination bands, while in the MIR, discriminative features were found in the Amide A and Amide B regions. The ability of optical spectroscopy to differentiate between various viral infections remains an open research question. Further investigation is needed, particularly concerning the use of viral transport media with more consistent and reproducible spectral profiles suitable for infrared analysis. This study also highlights the need for highly sensitive instruments when detecting viral presence in wet samples using the ATR method. The limitations of currently available low-cost MIR detectors, particularly their signal-to-noise ratio and stability, pose a barrier to widespread deployment of MIR-based POC systems. Therefore, advancements in the development of stable, cost-effective MIR detectors are essential to fully realize the potential of optical spectroscopy in real-world point-of-care diagnostics.

## Data Availability

The data that support the findings of this study are available from the corresponding author upon reasonable request.
